# Metabolic effect of low fluoride levels in the islets of NOD mice: integrative morphological, immunohistochemical, and proteomic analyses

**DOI:** 10.1590/1678-7757-2023-0036

**Published:** 2023-06-05

**Authors:** Juliana Sanches TREVIZOL, Aline DIONIZIO, Aislan Quintiliano DELGADO, Talita Mendes Oliveira VENTURA, Caroline Fernanda da Silva RIBEIRO, Laura RIBEIRO, Nathalia Rabelo BUZALAF, Tânia Mary CESTARI, Ana Carolina MAGALHÃES, Maiko SUZUKI, José Roberto BOSQUEIRO, Marília Afonso Rabelo BUZALAF

**Affiliations:** 1 Universidade de São Paulo Faculdade de Odontologia de Bauru Departamento de Ciências Biológicas Bauru SP Brasil Universidade de São Paulo, Faculdade de Odontologia de Bauru, Departamento de Ciências Biológicas, Bauru, SP, Brasil.; 2 Universidade Estadual Paulista Instituto de Biociências Botucatu São Paulo Brasil Universidade Estadual Paulista, Instituto de Biociências, Botucatu, São Paulo, Brasil.; 3 The Dental College of Georgia Department of Oral Biology and Diagnostic Sciences Augusta Georgia United States The Dental College of Georgia, Department of Oral Biology and Diagnostic Sciences, Augusta, Georgia, United States.; 4 Universidade Estadual Paulista Faculdade de Ciências Departamento de Educação Física Bauru São Paulo Brasil Universidade Estadual Paulista, Faculdade de Ciências, Departamento de Educação Física, Bauru, São Paulo, Brasil.

**Keywords:** Proteomics, Islet, Diabetes, Fluoride, NOD

## Abstract

Fluoride (F) has been widely used to control dental caries, and studies suggest beneficial effects against diabetes when a low dose of F is added to the drinking water (10 mgF/L).

**Objectives:**

This study evaluated metabolic changes in pancreatic islets of NOD mice exposed to low doses of F and the main pathways altered by the treatment.

**Methodology:**

In total, 42 female NOD mice were randomly divided into two groups, considering the concentration of F administered in the drinking water for 14 weeks: 0 or 10 mgF/L. After the experimental period, the pancreas was collected for morphological and immunohistochemical analysis, and the islets for proteomic analysis.

**Results:**

In the morphological and immunohistochemical analysis, no significant differences were found in the percentage of cells labelled for insulin, glucagon, and acetylated histone H3, although the treated group had higher percentages than the control group. Moreover, no significant differences were found for the mean percentages of pancreatic areas occupied by islets and for the pancreatic inflammatory infiltrate between the control and treated groups. Proteomic analysis showed large increases in histones H3 and, to a lesser extent, in histone acetyltransferases, concomitant with a decrease in enzymes involved in the formation of acetyl-CoA, besides many changes in proteins involved in several metabolic pathways, especially energy metabolism. The conjunction analysis of these data showed an attempt by the organism to maintain protein synthesis in the islets, even with the dramatic changes in energy metabolism.

**Conclusion:**

Our data suggests epigenetic alterations in the islets of NOD mice exposed to F levels comparable to those found in public supply water consumed by humans.

## Introduction

Fluoride (F) is widely used worldwide to control dental caries, mainly administered by fluoridated water and dentifrices.^1 , 2^ Fluoridation of public supply water is considered one of the 10 great public health achievements of the 20^th^ century^3^ due to its safety and good cost-benefit ratio.^4^ To achieve maximum protection against dental caries and avoid side-effects, F intake must be within the appropriate levels.^5^

Besides the beneficial effects of F to control dental caries, a series of animal studies conducted by our group has suggested that treatment with water containing 10 mgF/L (equivalent to 1–2 mgF/L for humans) could have beneficial effects against diabetes.^6 - 9^ This is relevant from a public health perspective, considering the global burden of this disease, which is estimated to directly cause 1.6 million deaths per year, and understanding it as a pandemic that poses increasing social and economic challenges.^10^

In one of our studies, male rats with streptozotocin-induced diabetes treated with fluoridated water for 22 days showed increased insulin sensitivity. This increase was attributed to impaired glucose metabolism in muscle, which could shift glucose metabolism to the liver, recruiting GRP-78 to the plasma membrane due to endoplasmic reticulum (ER) stress caused by F. In the membrane, GRP-78 complexes with ERj3P increased upon exposure to F, leading to increased insulin sensitivity.^6^

In more recent studies, we used non-obese diabetic (NOD) mice, which constitute a recognized model to analyze the mechanisms underlying type 1 diabetes (T1D) and preventive agents for this disease.^11^ In male NOD mice that received fluoridated water for 21 days, plasma glucose levels decreased and the percentage of β-cell function increased. Liver proteomic analysis showed an increase in antioxidant enzymes, such as glutathione S-transferase (GST).^7^ It has been suggested that lower expression in the islets of genes related to reactive oxygen species (ROS) metabolism, including several members of the GST family, contributes to β-cell injury and the development of diabetes in spontaneously diabetic biobreeding (BB) mice. Moreover, administration of antioxidants reduces the degree of insulitis and delays the development of diabetes in these animals.^12^ Thus, it was hypothesized that the reduced plasma glucose levels found in NOD mice after exposure to F could be due to increased antioxidant levels in the islets. Moreover, F is widely known to cause changes in energy metabolism.^7 , 9 , 13 , 15^ Recently, the F showed to reduce phosphoenolpyruvate carboxykinase (PEPCK) in the liver of NOD mice. The reduction of this enzyme, which positively regulates gluconeogenesis, can increase glucose uptake and also potentially contribute to explain, at the molecular level, the beneficial effects of low doses of F in reducing plasma glucose levels.^9^ However, the effect of F on the protein profile of islets has never been evaluated; therefore, this is the central aim of this study.

## Methodology

### Animals, fluoride treatment, and islets isolation

The experimental protocol was approved by the Animals Ethics Committee of Bauru School of Dentistry, University of São Paulo (CEUA-Proc. 013/2017). In total, 42 six-week-old female NOD mice were randomly divided into two groups, considering the concentration of F (as sodium fluoride) in the drinking water administered *ad libitum* to the animals for 14 weeks: 0 (control) or 10 mgF/L. This concentration of F simulates human consumption of water from the public supply, since the metabolism of F by rodents is 5–10 times faster when compared with humans.^16^ During the experimental period, animals received low F (~2 mg/kg) and normocaloric diet *ad libitum* . Female NOD mice were selected because they develop insulitis and diabetes to a higher degree than males. The 14-week treatment period was selected because insulitis starts at two to four and five to seven weeks in females and males, respectively, and hyperglycemia develops from 12 to 30 weeks.^11^

The animals were kept in a temperature and humidity controlled room with a 12-h light/dark cycle. The animals were euthanized at the end of the experimental period (exposure to CO_2_ followed by decapitation). The pancreas was collected for morphological and immunohistochemical analyses (n=12 animals per group). The collagenase digestion was used to isolate islets, as previously described,^17 - 19^ for proteomics analysis (n=9 animals per group). Islets from three animals per group were pooled to obtain biological triplicates for proteomic analysis. In addition to plasma fluoride, plasma glucose, and plasma insulin analysis were tested as previously described.^9^

### Morphological and immunohistochemical analyses

Pancreatic tissues were fixed in 10% formalin and embedded in paraffin. Then, 5-µm sections were fixed on slides, deparaffinized, and stained with hematoxylin and eosin (H&E). Morphological changes were recorded by a BX43 Upright optical microscope (Olympus Corporation Microscopes, Tokyo, Japan) with a camera attached, using the Olympus CellSens 1.14 software to capture and view the images. Photomicrographs were randomly divided into 10 fields and analyzed using the ImageJ 1.8.0 software. Values were expressed as the percentages of total pancreas area, total islet area and β-cells.

Immunohistochemical reactions were performed on formalin-fixed tissue slides, as previously described.^20^ The slides were deparaffinized, rehydrated, and incubated with peroxidase reagent, and then incubated with anti-insulin and anti-glucagon monoclonal antibodies (Santa Cruz Biotechnology, Inc.), as recommended by the manufacturer (Dako). Acetylated histone H3 (acH3K14) labeling was performed using rabbit anti-human acH3K14 antibodies (Cat. no. ab52946), following the manufacturer’s instructions. The tissue sections that were immunostained and H&E-stained were analyzed using a ScanScope model T3 (Aperio Technnologies, Leica Biosystems, USA) with 20x or 40x magnification. Individual slides were scanned and the area of the pancreas containing islets was selected manually. The percentage of inflammatory infiltrate was determined from the total area of each islet and the area of infiltrate present in the islet. For immunostained slides, the positive pixel-counting algorithm (PPCA) was used, with automatic quantification to obtain the percentage of labeled islets per animal, using the Image Scopus program version 12.4.0.^21^ The positive percentage of antigen-antibody reactivity was measured from the positive brown color in the islets, obtained from the total positive pixel color divided by the total number of pixels.

### Proteomics and bioinformatics analyses

The microtubes containing the islets in Hank’s buffer with protease inhibitor (10% v/v) were centrifuged for 5.000 g at 4°C for 5 min. After sediment formation, the supernatant was discarded, and the pellet was used for analysis. Extraction was performed using a solution with 6 M urea, 2 M thiourea prepared in 50 mM NH_4_HCO_3_ buffer (AMBIC) pH 7.8 (1:1 v/v). The samples were vortexed at 4°C for 10 min, sonicated for 5 min (under ice) and centrifuged at 20.817 g at 4°C for 10 min. This process was repeated twice. The samples were quantified using the Bradford method.^22^

After protein extraction, AMBIC (1.5 volume of sample) was added and samples were concentrated (~150 µL) and centrifuged at 4.500 g at 4°C. Reduction was performed by adding 5 mM dithiotreitol (DTT) (40 min at 37°C) and alkylation by adding 10 mM iodacetamide (IAA) (in the dark, 30 min). Then, 5 µL of AMBIC were added. The samples were digested by the addition of 2% (w/w) trypsin (Thermo Scientific, Rockford, USA) and incubated overnight at 37°C. Later, 5% trifluoroacetic acid (10 µL) was added, the samples were purified and desalinated using C18 Spin columns (Thermo Scientific^®^, Rockford, Illinois, USA) and then resuspended in a solution with 3% acetonitrile (ACN) and 0.1% formic acid.

The Xevo G2 mass spectrometer (Waters) coupled with the nanoACQUITY system (Waters) for peptide analysis and the ProteinLynx Global Server (PLGS) software program version 3.03 was used to process and search for continuous LC-MSE data, as previously reported.^9 , 14^ Peptides were identified using the programs ion counting algorithm and a search of the updated Mus musculus database (UniProtKB). PLGS detected difference in expression between the groups using the t-test (p<0.05). The CYTOSCAPE^®^ software platform (Java^®^) was used to construct molecular interaction networks between the identified proteins, supported by the ClusterMarker^®^ and ClueGO applications.

Mass spectrometry proteomics data were deposited in the ProteomeXchange consortium (http://proteomecentral.proteomexchange.org) via the PRIDE partner repository^23^ with the dataset identifier PXD031865.

### Statistical Analyses

For the analysis of morphological and immunohistochemical data, the GraphPad InStat program (version 3.0 for Windows, GraphPad Software Inc., La Jolla, CA, USA) was used. The data obtained were subjected to verification of normal distribution (Kolmogorov-Smirnov test) and homogeneity (Bartlett’s test). Once these parameters were evaluated, data were analyzed by unpaired t-tests (p<0.05).

## Results

### Morphological and immunohistochemical data

**Figure 1:**
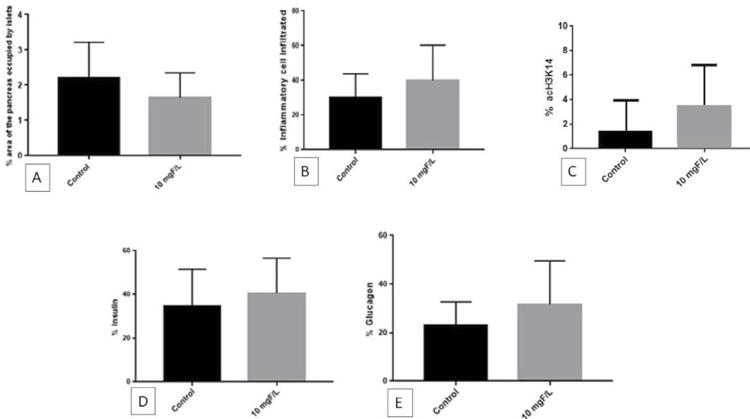
Mean percentage: (A) area occupied by islets; (B) inflammatory cell infiltrate; (C) acetylated histone H3 (acH3K14) labeling; (D) insulin labeling; and (E) glucagon labeling in the pancreas of female NOD mice treated with water containing 10 mgF/L or not (control) for 14 weeks. Bars show SD. The differences between the groups were not significant (unpaired t-tests; p>0.05). n=12, except for acH3K14 (n=5)

**Figure 2:**
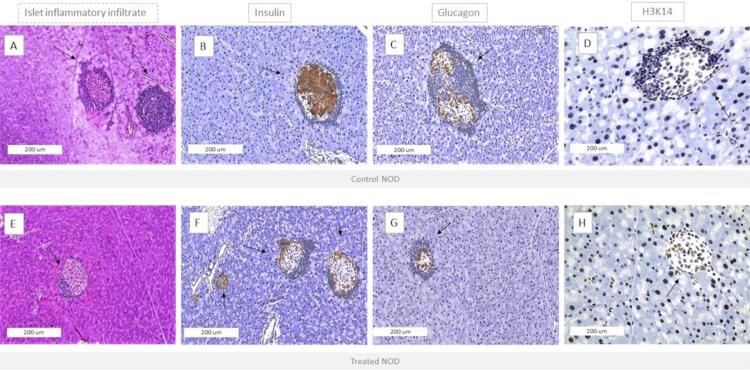
Pancreas sections from 20-week-old female NOD mice treated with deionized water (control) (A, B, C, and D) or water containing 10 mgF/L (as NaF) for 14 weeks (E, F, G, and H). Images A and E show H&E staining, with the presence of inflammatory infiltrate in the islets. Images B, C, F, and G show the samples stained (in brown) for insulin (B and F) and glucagon (C and G) and counterstained with hematoxylin. Images D and H show acH3K14 labeled in the pancreas. The images were generated at higher resolution (20x). Only Images D and H were generated at 40x, using the Image Scopus software

The mean percentages of pancreatic areas occupied by islets (±SD) were 2.22±0.99% and 1.64±0.71% ( Figure 1A ) for the control and treated groups, respectively, with no significant differences between them (p=0.25). Regarding pancreatic inflammatory infiltrate, the mean percentages for the control and treated groups were 32.4±10.9% and 43.8±16.4%, respectively, with no significant differences between them (p=0.06) ( Figure 1B ). The mean percentages of acH3K14-labeled cells were 1.55±2.76% and 3.64±3.59% for the control and treated groups, respectively, with no significant differences between them (p=0.23) ( Figure 1C ). The mean percentages of insulin-labeled cells were 34.4±17.2% and 40.6±16.0% for the control and treated groups, respectively ( Figure 1D ). The mean percentages of glucagon-labeled cells were 23.2±9.5% and 31.7±18.0% for the control and treated groups, respectively ( Figure 1E ). Although the treated group had a higher percentage of insulin-labeled and glucagon-labeled cells, a higher percentage of inflammatory infiltrate, and a higher degree of histone H3 acetylation than the control group, the differences were not significant (p=0.36 for insulin, p=0.24 for glucagon, p=0.06 for inflammatory infiltrate, and p=0.23 for acH3K14). The figure 2A-F shows representative images of the inflammatory infiltrate in the islets, as well as insulin and glucagon labeling.

### Proteomic analysis

**Figure 3:**
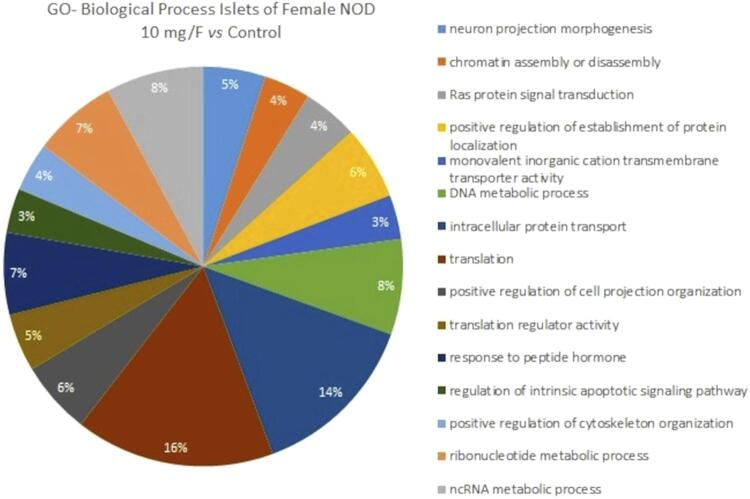
Functional distribution of proteins identified with differential expression in the islets of female NOD mice treated with 10 mgF/L vs. the control group (0 mgF/L). Protein categories were based on the GO “Biological Process” annotation. Significant terms (kappa=0.04) and distribution according to the percentage of number of genes. Proteins accession number was provided by UNIPROT. Gene ontology was evaluated according to the ClueGo® plugins in Cytoscape® 3.8.0.24,25

Proteomic analysis of the islets identified 385 proteins with differential expression between the groups. The number of proteins identified exclusively in the treated and control groups was 169 and 118, respectively. Among the proteins identified uniquely in the control group, several are related to energy metabolism, such as alpha-enolase; malate dehydrogenase, cytoplasmic; acetyl-CoA acetyltransferase, mitochondrial; succinate dehydrogenase [ubiquinone] flavoprotein subunit, mitochondrial; and phosphoenolpyruvate carboxykinase (PEPCK). Moreover, in the F-treated group, there were 45 proteins with increased expression, including histones and histone acetyltransferases, and 53 proteins with reduced expression compared with the control group (0 mgF/L) ( Supplementary Table 1 ). The biological processes regarding functional classification affected the most by F-treatment were translation (16%), intracellular protein transport (14%), ncRNA metabolic process (8%), DNA metabolic process (8%), ribonucleotide metabolic process (7%) and response to peptide hormone (7%) ( Figure 3 ).

**Figure 4:**
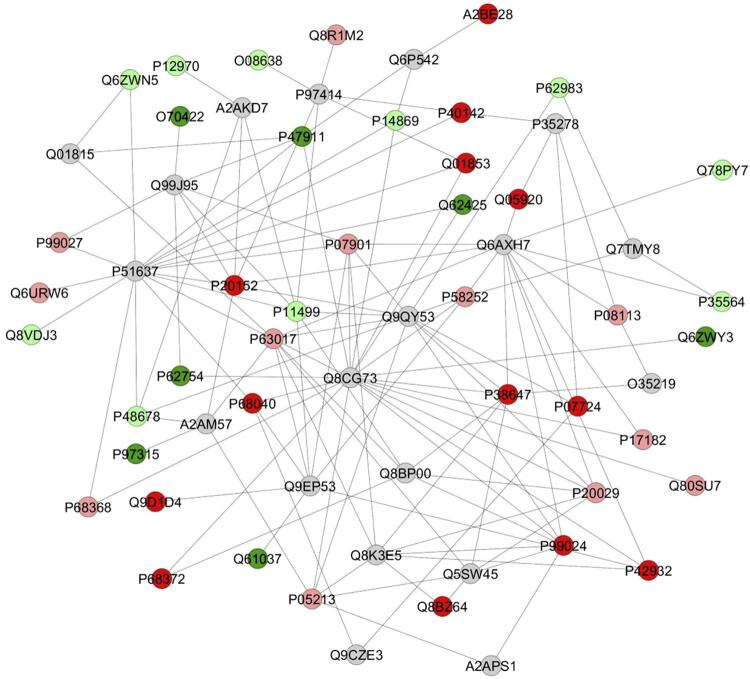
Subnetwork generated by ClusterMarker® for the comparison between treatment with 10 mgF/L and the control group (0 mgF/L). The node color shows the differential expression of the protein with its access code, obtained from the UniProt protein database (http://www.uniprot.org/). Dark green and dark red nodes represent proteins identified exclusively in the treated and control groups, respectively. Light red and light green nodes represent down- and up-regulated proteins, respectively, in the treated group compared with the control group. Gray nodes represent interaction proteins offered by CYTOSCAPE®, which were not identified in this study

The network generated by ClusterMarker2 when comparing the expression of altered proteins in the treated group (10 mgF/L) versus the control group (0 mgF/L) suggests that many proteins interact with nephrocystin-1 (Q9QY53), caveolin (P51637), receptor for activated C kinase 1 (Q8CG73), IQ calmodulin-binding motif-containing protein 1 (Q8BP00), [histone H3]-lysine(27) N-trimethyltransferase (Q6AXH7) and hamartin (Q9EP53) ( Figure 4 ).

**Figure 5:**
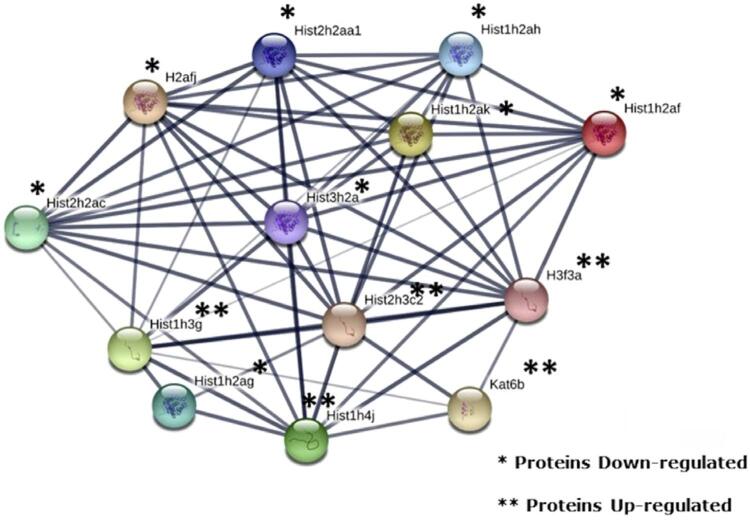
Analysis in the stringApp® application (CYTOSCAPE® 3.9.0) with 138 proteins found in the treated group vs. the control group (0 mgF/L) related to different metabolic pathways. The internal stains of the light green and light pink nodes represent up- and down-regulated proteins, and dark green and dark red nodes represent proteins unique to the treated and control groups, respectively. Around the nodes, the colors in the table show which gene ontology, KEEG or Reactome pathways changed. The FDR (false discovery rate) corresponds to the statistical analysis used. p<0.05

To identify the biological processes or metabolic pathways (categories: gene ontology, KEEG pathways, and Reactome pathways) involved in the proteins/genes with altered expression, the stringApp^®^ bioinformatics analysis (CYTOSCAPE^®^ 3.9.0) was used ( Figure 5 ). The lines connecting the gene nodes represent the protein-protein interaction, but are not relevant at this point, since the results of functional enrichment analysis related to the metabolic processes and/or pathways were the focus; they are represented in the Description column. The greater significance of each category is given by the false discovery rate (FDR): the lower this value, the more enriched the process or metabolic pathway. In the STRING network, outer node colors correspond to the colors of the respective processes or metabolic pathways (category) of which that gene/protein is a part (represented in the “color” column). The interior of the nodes represents the expression of proteins/genes (light green and light pink represent up- and down-regulated proteins, respectively, and dark green and dark red represent proteins exclusive to the 10 mgF/L and control groups, respectively). Most proteins or genes identified in the pancreatic islets as belonging to metabolism-related processes or pathways are down-regulated in the F-treated group or exclusive to the control group, which suggests a reduction in overall metabolism in the presence of fluoride (10 mgF/L). These proteins are mainly related to DNA metabolic process (39 genes), metabolism of amino acids and derivatives (24 genes), ATP metabolic process (30 genes), glycolysis/gluconeogenesis (18 genes), HATs acetylate histones (12 genes) and carbohydrate metabolic process (33 genes) ( Figure 5 ).

## Discussion

This is the first study to evaluate the proteomic profile of the islets of NOD mice exposed to F levels comparable with those found in public supply water consumed by humans. The results of plasma F, plasma glucose, and plasma insulin were previously described.^9^ Briefly, exposure to F significantly increased plasma fluoride levels, reduced plasma glucose levels by 20%, and had no effect on plasma insulin. The reduction in plasma glucose levels in NOD mice exposed to water containing a low dose of F (10 mg/L) was also observed in another study by our group.^7^ This may be relevant for public health, since fluoridation of public water, a globally recognized measure to prevent dental caries,^5^ may also play a role in diabetes prevention. However, the possible mechanisms involved in this beneficial effect of F against diabetes, if confirmed, are unknown. A possible explanation could be the well-known effects of F on impaired energy metabolism,^7 , 9 , 13 - 15^ which was also observed in this study ( Figure 5 ). Glycolytic enzymes (alpha-enolase), the Krebs cycle (malate dehydrogenase, cytoplasmic), beta-oxidation (acetyl-CoA acetyltransferase, mitochondrial), and oxidative phosphorylation (succinate dehydrogenase [ubiquinone] flavoprotein subunit, mitochondrial) were absent after exposure to F, as shown in Figure 5 , with a predominance of the control group, especially in ATP metabolic process. It has been known since the 1940s that F inhibits enolase,^26^ a key glycolytic enzyme that also performs several non-glycolytic “moonlighting” functions,^27 , 28^ such as glucose homeostasis, by regulating PEPCK expression. We recently reported that NOD mice exposed to water containing 10 mgF/L do not show PEPCK expression in the liver.^9^ This enzyme is a positive regulator of gluconeogenesis and, when down-regulated, increases glucose uptake, which, in turn, reduces plasma glucose. Interestingly, in this study, PEPCK was also absent in the islets of the F-treated group, confirming our previous findings in liver and muscle and indicating the decrease in PEPCK as the probable mechanism by which low F levels reduce plasma glucose.

Another plausible reason for the beneficial effect of F against diabetes was the increase in the antioxidant defense in the islets. It is known that genes involved in the anti-oxidative defense are less expressed in spontaneously diabetic lymphopenic biobreeding (BB) rats, which is implicated in β-cell damage and the development of diabetes in these animals. This can be countered by administration of antioxidants, such as N-acetyl-cysteine.^12^ In previous studies, we observed an increase in some antioxidant enzymes, such as GST in the liver of NOD mice exposed to water containing 10 mgF/L.^7 , 9^ We then hypothesized that increased expression of antioxidant enzymes in the islets of NOD mice after exposure to F could delay the development of diabetes, this was one of the reasons for this study. However, this was not the case. In this study, antioxidant proteins, such as GST and peroxiredoxin, were found exclusively in the control group. It is important to highlight that, in the morphological and immunohistochemical analyses, we found no significant differences between the groups in insulin and glucagon-labeled cells, which shows that changes caused by F at this stage were present only at the molecular level. The lack of significant differences in the percentage of insulin- labeled, glucagon-labeled and acH3K14-labeled cells in the morphological and immunohistochemical analysis between the control and treated groups reinforces the safety of optimal water fluoridation.

Protein disulfide isomerase 6 (PDIA6) reduced by more than 3-fold after exposure to F. Studies have shown that low expression of this chaperone, necessary for S-S bond formation and proper insulin, can cause accumulation of unfolded proteins, trigger the unfolded protein response (UPR), and generate ER stress in β-cells.^29^ The reduction in PDIA6 may have been compensated by the increase in other chaperones that might have allowed proper protein folding, such as PDIA3 (8-fold), calnexin (7-fold; Figure 4 ) and calreticulin (2-fold).

**Figure 6:**
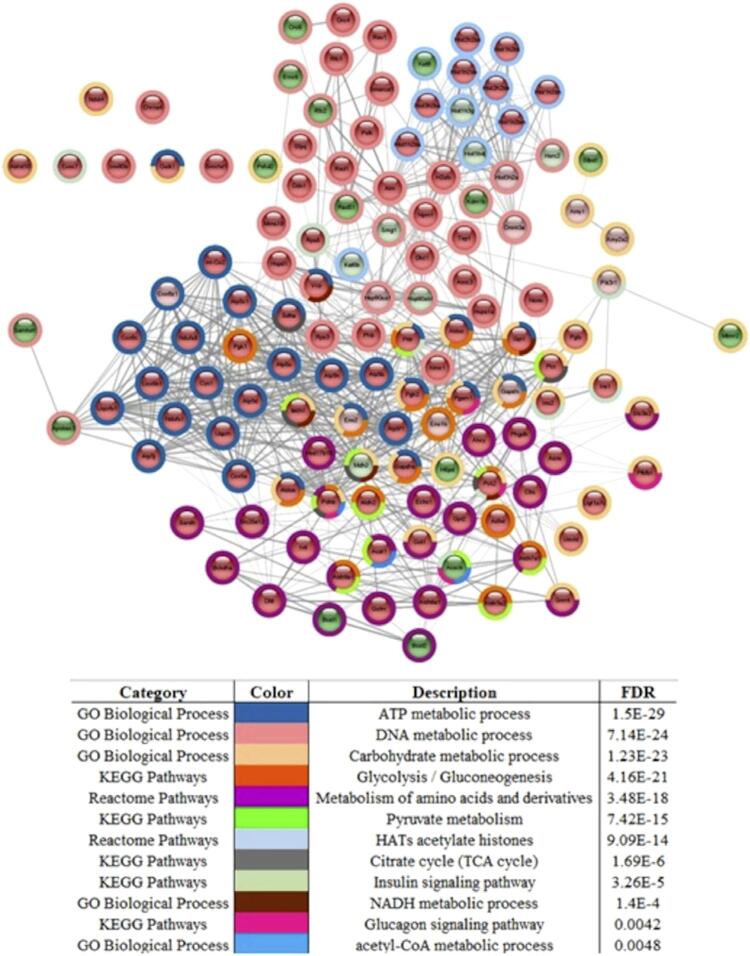
Protein-protein interaction network of histones and histone acetyltransferases. STRING database version 11.0 (https://string-db.org/). The following proteins are present in the network: Hist1h2ak (Q8CGP7) – Histone H2A type 1-K; Hist1h2ah (Q8CGP6) – Histone H2A type 1-H; Hist1h2af (Q8CGP5) – Histone H2A type 1-F; Hist2h2ac (Q64523) – Histone H2A type 2-C; Hist1h4j (P62806) – Histone H4, Hist1h2ag (C0HKE1) – Histone H2A type 1-B; Hist1h3g (P68433) – Histone H3.1; Hist2h3c2 (P84228) – Histone H3.2; Hist2h2aa1 (Q6GSS7) – Histone H2A type 2-A; Hist3h2a (Q8BFU2) – Histone H2A type 3; H2afj (Q8R1M2) – Histone H2A.J; Kat6b (Q8BRB7) – Histone acetyltransferase KAT6B; and H3f3a (P84244) – Histone H3.3

Environmental factors can cause epigenetic changes that regulate gene expression and influence immune cell function. Therefore, epigenetics offers several molecular mechanisms that help explain the environmental effects involved in the development of autoimmune diabetes. The three main epigenetic changes include DNA methylation, histone modification, and microRNA alteration.^30^ The most remarkable finding of this study was the expressive change in the expression of histones and histone modifying proteins, as well as in other proteins involved in transcription and translation (such as ribosomal proteins). Dysregulation of histone acetylation, under the control of histone deacetylases (HDACs) and histone acetyltransferases (HATs), is involved in the pathogenesis of diabetes.^31^ Histone acetylation has been shown to maintain insulin secretion during cytokine insult and prevent NF-κB induced apoptosis in a β-cell line and islets of newborn rats.^32^ In vitro and in vivo studies have shown that F up-regulates NF-kB gene expression,^33^ a key mediator of inflammatory responses that induces the expression of several pro-inflammatory genes.^34^ This is consistent with increased inflammatory infiltrate in the islets of the F-treated group ( Figure 1B ). Another noteworthy finding of this study was the large increase in histones H3.1 and H3.2 (100-fold) and H4 (more than 60-fold), combined with the 6-fold increase in histone acetyltransferase KAT6B and histone acetyltransferase KAT8 ( Figures 5 and 6 ), which was found exclusively after F-treatment. Moreover, the percentage of histone H3 acetylation was more than 2-fold higher in the group exposed to F when compared with the control group, although the difference did not reach statistical significance ( Figure 1C ). It is important to highlight that we evaluated acetylation of H3K14, but not other targets, because H3K14 is one of the acetylation targets of KAT6B and KAT8. During histone acetylation, an acetyl functional group is transferred from acetyl-CoA to the histone. In this study, pyruvate carboxylase (PC), mitochondrial and PEPCK were not found in the F-treated group. These enzymes participate in important anaplerotic pathways in the Krebs cycle, allowing the formation of oxaloacetate from phosphoenolpyruvate (PEP), and are therefore essential for replenishing acetyl-CoA. Moreover, as aforementioned, F also reduces glycolysis. These findings show that F reduces the formation of acetyl-CoA in several ways, reducing glycolysis and decreasing the anaplerotic formation of oxaloacetate from PEP. The large increase in the expression of histones and HATs in this study in the presence of F might have been an attempt to maintain translation at low acetyl-CoA levels. Increased expression of histones has been associated with increased lifespan in yeast, since it maintains the fundamental chromatin structure.^35^ A recent study showed that 5 mM F increases the activity of histone acetyltransferases in LS8 cells. However, the dose used was much higher than in this study.^36^ Prelamin-A/C (P48678) increased 33-fold after exposure to F. This protein plays an important role in nuclear assembly and chromatin organization (UNIPROT). Its increase may be also related to the organisms attempt to maintain translation in the presence of F. Recent studies have shown that F-induced hypermethylation in key genes is involved in distinct toxicity effects.^37 - 39^

Staphylococcal nuclease domain-containing protein 1 (Q78PY7) increased more than 3-fold after exposure to F ( Supplementary Table 1 ; Figure 4 ). This enzyme is an endonuclease that mediates miRNA decay of protein-free and AGO2-loaded miRNAs. It functions as a transcriptional coactivator for STAT5 (UNIPROT), which has been shown to interact with various HATs, including CBP and SRC-1, and recruit HDACs, thus playing a role in transcription.^31^

Many proteins with altered expression in the subnetwork ( Figure 6 ) interacted with [histone H3]-lysine (27) N-trimethyltransferase (Q6AXH7), also known as histone methyltransferase EZH2 (enhancer of zeste homolog 2). This enzyme, a master of epigenetic regulation, suppresses gene transcription mainly by catalyzing the trimethylation of histone H3 at lysine 27 (H3K27me3). It also alters the metabolic patterns of tumor cells by distinct pathways, involving amino acids, glucose, lipids, and metabolism.^40^ Acetylation of EZH2 enhances its stability and function and requires acetyl-CoA,^40^ which can reduce in the presence of F, as aforementioned. This might have compromised the stability of histone methyltransferase EZH2. It is important to note that among the biological processes most affected by F are translation (16%) and ncRNA metabolic process (8%), which may be possibly related to the epigenetic role of F in histone methyltransferase EZH2 and in HATs. Further studies using appropriate tools are needed to evaluate F-induced epigenetic changes in islets.

## Conclusion

Our data confirm previous findings in the liver and muscle, pointing the decrease in PEPCK as a possible mechanism underlying the reduction in plasma glucose levels by F. Moreover, our results suggest F-induced epigenetic changes in the islets, since we found large increases in histones H3 and, to a lesser extent, in HATs, concomitant with a decrease in enzymes involved in the formation of acetyl-CoA, especially metabolic enzymes involved in glycolysis. The conjunction analysis of these data shows the organisms attempt to maintain protein synthesis in the islets, even with the dramatic changes in energetic metabolism typically induced by F.
